# Speckle-tracking echocardiography in comparison with ejection fraction for prediction of cardiovascular mortality in patients with end-stage renal disease

**DOI:** 10.1093/ckj/sfaa161

**Published:** 2021-01-19

**Authors:** Janna Terhuerne, Merel van Diepen, Rafael Kramann, Johanna Erpenbeck, Friedo Dekker, Nikolaus Marx, Jürgen Floege, Michael Becker, Georg Schlieper

**Affiliations:** 1 Division of Nephrology and Clinical Immunology, Medical Faculty RWTH Aachen University, Aachen, Germany; 2 Department of Clinical Epidemiology, Leiden University Medical Center, Leiden, The Netherlands; 3 Department of Cardiology, Medical Faculty RWTH Aachen University, Aachen, Germany; 4 Center for Nephrology, Hypertension, and Metabolic Diseases, Hannover, Germany

**Keywords:** cardiovascular, dialysis, echocardiography, ESRD, prognosis

## Abstract

**Background:**

Cardiovascular disease is the major cause of death in end-stage renal disease (ESRD). To develop better means to assess cardiovascular risk in these patients, we compared conventional echocardiography-derived left ventricular ejection fraction (EF) with the novel method of 2D speckle-tracking echocardiography to determine cardiac strain.

**Methods:**

Predictive performances of conventional EF and speckle-tracking echocardiography-derived global longitudinal strain (GLS) were compared using receiver-operator curve (ROC) analyses and calibration by calibration plots. We also took into account other known cardiovascular risk factors through multivariable logistic regression analysis.

**Results:**

The study comprised 171 ESRD patients (mean age 64 years, 64% male) on maintenance dialysis therapy (93% haemodialysis, 7% peritoneal dialysis) for an average period of 39 months. During 2.1 years of follow-up, 42 patients (25%) died from cardiovascular disease. ROC analysis of GLS resulted in an area under the curve of 0.700 [95% confidence interval (CI) 0.603–0.797] compared with an area under the curve of EF of 0.615 (95% CI 0.514–0.716) (P = 0.059 for difference). The total absolute deviation between predicted and observed outcome frequencies obtained by calibration plots were 13.8% for EF compared with only 6.4% for GLS. Best results of ROC analysis (area under the curve = 0.759; P = 0.06), calibration and goodness-of-fit (χ^2^ = 28.34, P ≤ 0.0001, *R*^2^ = 0.25) were achieved for GLS added to a baseline model consisting of known cardiovascular risk factors in a multivariate regression analysis.

**Conclusions:**

In summary, in chronic dialysis patients, GLS is a more precise predictor of cardiovascular mortality than conventional echocardiography-derived EF.

## INTRODUCTION

Chronic kidney disease (CKD) patients suffer from an up to 30 times increased risk of cardiovascular mortality compared with the general population and are more likely to die of cardiovascular disease than reach the dialysis stage. A major clinical problem is that assessing cardiovascular disease in advanced CKD patients is unreliable, and non-invasive gold standards are lacking [1–3]. In addition, cardiovascular causes of death in advanced CKD differ from the normal population and mostly encompass heart failure and sudden cardiac death and to a lesser degree ischaemic heart disease [4–6]. In uraemic cardiomyopathy, cardiac fibrosis and capillary rarefaction are considered to contribute to cardiac dysfunction.

First subclinical changes upon imaging are common in the early stages of uraemic cardiomyopathy [7]. The present best-established method to assess ventricular dysfunction in general is contrast-enhanced magnetic resonance imaging, which, however, carries the disadvantage of high cost and need for a gadolinium-containing contrast agent. Speckle-tracking echocardiography strain imaging, also known as deformation imaging, is a relatively novel technique to objectively assess regional myocardial function, and it has been validated against magnetic resonance imaging in various clinical trials in the non-renal population [8, 9]. Although speckle-tracking echocardiography imaging has advantages compared with conventional echocardiographic determination of ejection fraction (EF) with respect to accurateness, cardiac load and heart rate independency, as well as reproducibility [10], conventional echocardiographic EF measurement still remains the most widely used diagnostic tool in CKD patients. This is partly attributable to the lack of adequate studies and reference values as dialysis patients are often excluded from clinical trials, as well as uncertainty concerning the pathophysiology of ureamic cardiomyopathy [11, 12]. The fact, that a large number of end-stage renal disease (ESRD) patients, even those with cardiac symptomatology, exhibit preserved EFs (i.e. >55%), yet at the same time represent the group of highest cardiovascular risk, indicates the urgent need for better techniques to stratify risk [13–15].

Out of many strain parameters, global longitudinal strain (GLS) has emerged as a significant prognostic marker for cardiovascular outcome in various study populations with subtle or apparent systolic dysfunction and cardiac abnormalities. Thus, patients suffering from heart failure or hypertrophic cardiomyopathy, patients getting revascularization therapy after myocardial infarction or patients suffering from diabetes, systemic sclerosis or amyloidosis with subclinical myocardial involvement have been shown to benefit from GLS determination as a prognostic marker [16–19].

Concerning CKD patients, results of a recent long-term study revealed that GLS represents a significant predictor of all-cause mortality in pre-dialysis and dialysis patients [20]. In a second recent study, left ventricular longitudinal strain had greater prognostic power concerning all-cause and cardiovascular mortality than conventionally determined EF in patients with advanced CKD [13]. An association between GLS and cardiac death and events was also shown in a study with haemodialysis patients when accounting for EF [21]. When comparing several strain parameters with routine echocardiographic parameters and clinical variables, we recently reported that speckle-tracking echocardiography is an effective method to detect ureamic cardiomyopathy [7]. Left ventricular longitudinal strain emerged as a risk factor for cardiovascular mortality and left ventricular circumferential strain for all-cause mortality, but the predictive performance of speckle-tracking echocardiography was not assessed. Based on the data from our recent study [7], we therefore used various predictive performance measures to find out how well GLS works as a predictor of cardiovascular mortality compared with conventional echocardiographic EF. In our comprehensive approach, we used different predictive models.

## MATERIALS AND METHODS

### Clinical assessment

In this study using data of our recent study [7], we focused on the predictive performance of a particular strain parameter, GLS, in comparison to EF. The study population consisted of prevalent dialysis patients. Echocardiography was performed in the Rheinisch-Westfälische Technische Hochschule Aachen (RWTH) Aachen University Hospital Department of Cardiology between January 2006 and December 2008. Data concerning demographic factors and medical history of these patients were obtained by chart review. The Ethics Committee of the RWTH Aachen University Hospital approved the study.

### Echocardiographic assessment

Echocardiographic imaging was performed using a digital ultrasound scanner (Vivid 7; GE Healthcare) with a 2.5-MHz transducer recording 2D loops from four left ventricular views (apical two-, three-, four-chamber and parasternal short-axis view). In haemodialysis patients, echocardiography was carried out on a non-dialysis day. EF determination and speckle-tracking analysis were performed offline using EchoPAC-PC software version 110.1.3 (GE Healthcare). End-diastolic and end-systolic volume-dependent endocardial border movement was manually traced and EF was calculated with the help of Simpson’s biplane method and with a frame rate from 50 to 90/s. Longitudinal strain was assessed from the three apical views leading to 18 segments, while parasternal short-axis view was used to gain circumferential and radial strain parameters using a six-segment model. Adequate image quality was ensured by the software mentioned above, as segments with insufficient tracking quality were excluded automatically [7].

### Follow-up

Follow-up information was obtained via the patients’ hospital charts, by telephone calls and with the help of the German death registry. The primary endpoint was cardiovascular death. Cardiovascular death was diagnosed when death was attributed to myocardial infarction, cardiogenic shock or stroke. Death occurring outside the hospital for which no other cause was specified was regarded as sudden cardiac death and included in the definition of cardiovascular death if the patient had known cardiovascular disease (i.e. prior myocardial infarction or chronic heart failure) and was not diagnosed with cancer [7].

### Statistical analysis

Descriptive statistics included mean value plus standard deviation for continuous variables and numbers with percentages for categorical variables. Missing values were handled through complete case analysis. The algebraic sign of EF was converted into negative values just to facilitate comparison with longitudinal strain parameters, whose negative values are caused by the fact that ventricular myocardium shortens along the longitudinal axis during systolic activity. To compare the predictive performance of the two continuous parameters (GLS and EF) for predicting cardiovascular mortality as binary outcomes, we applied univariate logistic regression analysis and assessed both discrimination and calibration.

Discrimination indicates how well a prognostic tool can discriminate subjects with and without the outcome. Discrimination was assessed by receiver-operator curve (ROC) analysis and presented as area under the curves (c-index) with corresponding 95% confidence intervals (CIs). Area under the curves range from 0.5 to 1, where 0.5 represents no discrimination and 1 represents perfect discrimination [22]. To investigate if there was a clinically meaningful superiority of GLS in predicting cardiovascular mortality, the two parameters’ discriminative ability was compared using a Chi-square test and P-value of the difference were presented. The major advantage of using area under the curves to compare predictive performance instead of single pairs of sensitivity and specificity values is the elimination of choice of a threshold value [23].

Next, calibration was assessed. Calibration indicates how well the predicted probabilities of a prognostic tool agree with the observed frequencies of the outcome. On the basis of probabilities obtained by logistic regression, calibration plots were created assessing the concordance of observed and predicted cardiovascular death rates. Predicted probabilities and observed frequencies of four quartiles of increasing predicted risk were compared and presented as bar charts.

To further investigate whether longitudinal strain adds predictive value beyond EF both parameters were added and compared with a baseline prediction model consisting of four relevant clinical prognostic factors of cardiovascular death in ESRD patients (i.e. age, sex, presence of diabetes mellitus and dialysis vintage). The number of clinical variables included was restricted by the observed outcome frequency (*n* = 42). Multivariate logistic regression analysis was performed using the (i) baseline model, (ii) baseline model + EF and (iii) baseline model + GLS. We used logistic regression because of short timeframe of follow-up. For all models, area under the curves and calibration plots were determined. In addition, goodness-of-fit was assessed by Chi-square test and Nagelkerke *R*^2^.

Finally, calibration-in-the-large, that is the difference in observed outcome frequency and average predicted probability in the total sample without division into quartiles, was computed for the univariate and multivariate analysis.

Statistical analyses were performed using SPSS version 22 (SPSS, Inc.). For ROC comparison SAS version 9.4 (SAS, Inc.) was used. A P-value of <0.05 was considered statistically significant.

## RESULTS

### Clinical characteristics

The study cohort consisted of 171 ESRD patients with appropriate image quality. Mean (SD) age was 64 ± 14 years with predominant male gender (65%) undergoing dialysis (93% haemodialysis, 7% peritoneal dialysis) for a mean length of 39 ± 55 months. Thirty-one patients had previously received a kidney transplant and had restarted dialysis therapy 4.6 ± 5.3 years (mean ± SD) before echocardiography was performed. The average follow-up was 2.1 ± 0.9 years (mean ± SD). The primary endpoint, cardiovascular death, was observed in 42 patients (24.6%) after 311 ± 302 days of follow-up (mean ± SD). The only missing data were dialysis vintage in 18 of 171 patients. Baseline characteristics are presented in Table 1.

**Table 1.** Baseline characteristics of the study population (*n* = 171)

Characteristics	Study population (*n* = 171)
Age, (mean ± SD), years	64 (±14)
Sex (men), *n* (%)	111 (65)
Body mass index, (mean ± SD), kg/m²	27 (±5)
Smokers, *n* (%)	43 (25)
Dialysis time, (mean ± SD), month	39 (±55)
Co-morbidities, *n* (%) Diabetes mellitus Type 1 or 2 Coronary heart disease STEMI Atrial fibrillation Heart failure Renal anaemia, *n* (%)	69 (40) 102 (60) 23 (13) 66 (39) 41 (24) 124 (73)
Underlying renal disease, *n* (%) Diabetic nephropathy Hypertensive nephrosclerosis Glomerulonephritis Polycystic kidney disease Interstitial nephritis Systemic disease^a^ Unknown	49 (29) 46 (27) 31 (18) 18 (11) 9 (5) 7 (4) 11 (6)
Laboratory values (mean ± SD) Haemoglobin, g/L S-potassium, mmol/L S-total calcium, mmol/L	109 (±16) 4.7 (±0.8) 2.2 (±0.3)
Medication, *n* (%) β-blockers ACE inhibitor Diuretics Phenprocoumon	132 (77) 74 (43) 102 (60) 35 (20)
Echocardiographic values (mean ± SD) EF, % GLS, %	49.6 (±14) −12.0 (±4)

STEMI, ST-segment elevation myocardial infarction; ACE, angiotensin-converting enzyme.

a Lupus erythematosus, sarcoidosis, rheumatoid arthritis, Wegener granulomatosis, Henoch–Schönlein purpura, Churg–Strauss arteritis and polyarteritis nodosa.

### Comparison of predictive performance of GLS and EF

**FIGURE 1:**
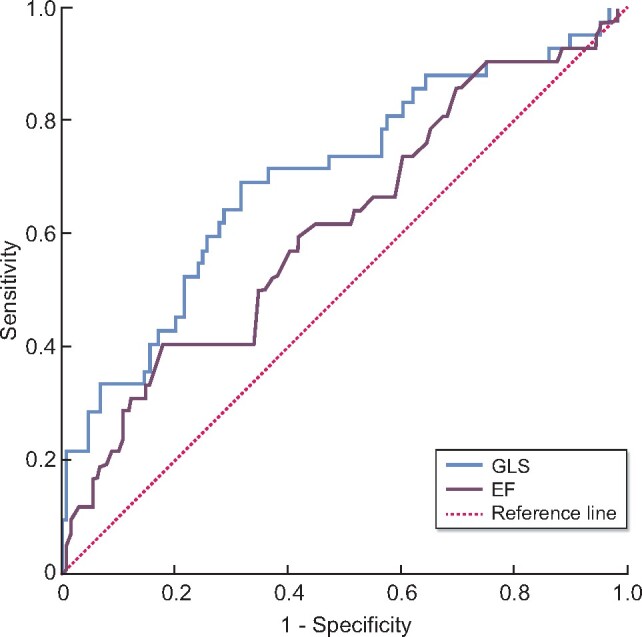
ROC curves showing the predictive performance of GLS and EF concerning cardiovascular mortality. GLS obtained a better predictive result (area under the curve: 0.700; 95% CI 0.603–0.797) than EF (area under the curve: 0.615; 95% CI 0.514–0.716). Longitudinal strain parameters show negative values as ventricular myocardium shortens along the longitudinal axis during systolic activity. For easier comparison, values of EF were modified by changing the algebraic sign.

Using ROC we analysed the predictive performance of GLS and EF. The area under the curve for GLS was 0.700 (95% CI 0.603–0.797) and thus higher than that of EF (0.615; 95% CI 0.514–0.716) (Table 2 and Figure 1). To compare the discriminative ability of the two methods, we made use of the ROC contrast method including a Chi-square test. The corresponding P-value of 0.059 when comparing GLS to EF (Table 3) supported the view that GLS may be a more useful clinical marker to predict cardiovascular mortality although this was only marginally significant.

**Table 2.** Areas under the curve of EF and GLS

	Area under the curve (95% CI)
EF	0.615 (0.514–0.716)
GLS	0.700 (0.603–0.797)

**Table 3.** Comparison of area under the curve values of different parameters

	P-value
EF versus GLS	0.0590
Baseline + EF versus baseline	0.1223
Baseline + GLS versus baseline	0.0600

To compare probabilities with the observed outcome frequencies, we turned calibration plots into bar charts. The increasing risk predicted by GLS and EF was divided up into four quartiles. We found lower discrepancy, i.e. better calibration, in the case of GLS (Figure 3, Supplementary data, Figures S1 and S2).

### Comparison of predictive performance of GLS and EF in addition to a baseline prediction model

To further investigate and compare predictive performances of GLS and EF, we added four prognostic clinical variables, namely age, sex, diagnosis of diabetes mellitus and dialysis vintage, into a baseline prognostic model. Systolic blood pressure was not associated with cardiovascular parameters and therefore not included in further analysis.

**FIGURE 2:**
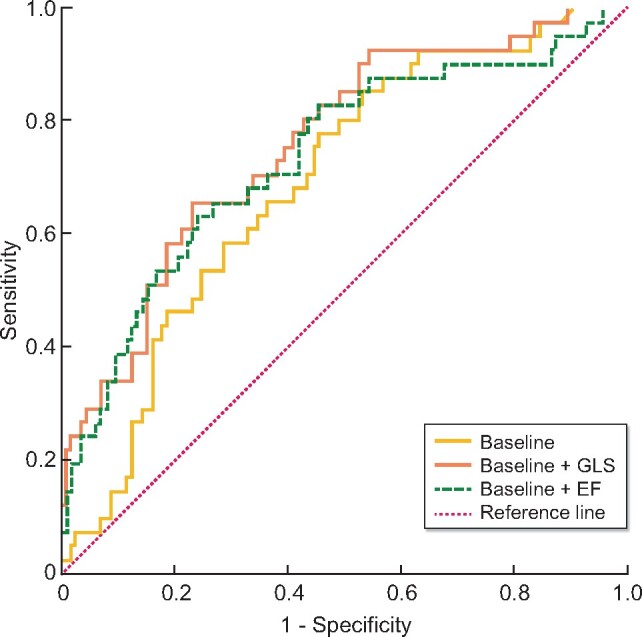
ROC curves representing predictive performance of the baseline model including four relevant clinical variables (age, sex, diabetes mellitus and dialysis vintage), as well as ROC curves of EF and GLS when added separately on top of the baseline model. ROC analysis of the baseline model itself yields an area under the curve of 0.690 (95% CI 0.600–0.780). Both, EF and GLS added separately to the baseline model improve the area under the curve. But, additive predictive value of GLS is higher (area under the curve: 0.759; 95% CI 0.673–0.845) than that of EF (area under the curve: 0.735; 95% CI 0.641–0.829). Baseline model = age, sex, diabetes mellitus and dialysis vintage

ROC analysis of all four parameters put together into one model yielded an area under the curve of 0.690 (95% CI 0.600–0.780), i.e. similar to GLS in terms of predictive performance and higher than that of EF (Tables 2 and 4). To analyse whether adding GLS and EF into the model increased predictive performance, we added both separately on top of the baseline model. The highest area under the curve was achieved by adding GLS to the baseline model (area under the curve = 0.759) with the lowest P-value (P = 0.06) when compared with the baseline model in isolation. The predictive value after adding EF to the baseline model was lower with an area under the curve of 0.735 and higher P-value (P = 0.12) (Tables 3 and 4, Figure 2).

**FIGURE 3:**
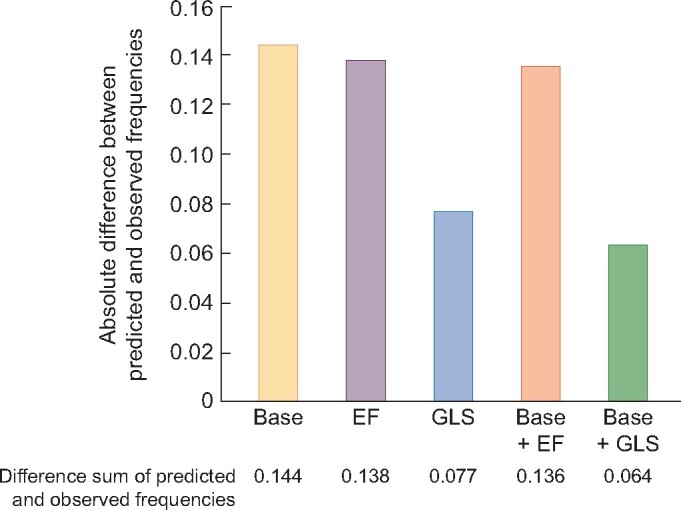
Bar chart summarizing the results of calibration plots. It shows the absolute difference between predicted and observed frequencies of cardiovascular mortality dependent on different predictors and their discriminative ability. GLS alone and GLS added to the baseline model (Base** **+** **GLS) carries the lowest absolute difference (0.077 and 0.064). EF causes higher discrepancy between observed and predicted mortality rates when used as predictor (0.138).

**Table 4.** Areas under the curve of EF and GLS in addition to other prognostic factors

	Area under the curve (95% CI)
Baseline	0.690 (0.600–0.780)
Baseline + EF	0.735 (0.641–0.829)
Baseline + GLS	0.759 (0.673–0.845)

Baseline = age, sex, diabetes mellitus, dialysis vintage.

Calibration plots showed that the best concordance of predicted and observed outcomes was achieved by adding GLS to the baseline model (Supplementary data, Figure S5).

Goodness-of-fit of the multivariate regression models, results of the Chi-square test and Nagelkerke *R*^2^ are presented in Table 5. The best result was as well achieved by GLS added to the baseline model (baseline + GLS: Chi-square = 28.34, P ≤ 0.0001, *R*^2^ = 0.25; baseline + EF: Chi-square = 21.90, P = 0.001, *R*^2^ = 0.19). Both, addition of GLS and EF, yielded a significant improvement but slightly better results were obtained by adding GLS.

**Table 5.** Results of goodness-of fit based on multivariate logistic regression model

	Chi-square	P-value	*R*² Nagelkerke
Baseline	13.509	0.009	0.123
Baseline + EF	21.903	0.001	0.194
Baseline + GLS	28.335	<0.0001	0.246

To evaluate model performance, we also computed calibration-in-the-large . We observed the lowest deviation in case of GLS alone and GLS added to the baseline model. These results support the interpretation that GLS represents a more potent predictor of cardiovascular death as compared with EF.

## DISCUSSION

In this study, we analysed the predictive performance of the strain parameter GLS and found it to outperform EF in predicting cardiovascular mortality in a cohort of dialysis patients with respect to discrimination, calibration and goodness-of-fit, both univariably and when added to other prognostic variables.

Both, EF and GLS, have been shown to be associated with outcome in patients with CKD. de Mattos *et al*. analysed EF within a cohort of 2718 dialysis patients waiting for renal transplantation. A reduced left ventricular EF (i.e. ≤40%) was associated with significantly higher mortality rates [24]. Payne *et al.* showed an association of reduced EF values (EF <30% and EF of 30–50%) with all-cause mortality in patients with non-dialysis-dependent CKD [25]. Longitudinal strain as a novel echocardiographic parameter and its correlation with different endpoints has been the subject of two studies in ESRD patients with preserved EF. The results revealed that less negative GLS values were significantly associated with an increased cardiovascular [26] and all-cause mortality [27]. Thus, even subtle myocardial changes are associated with increased mortality in advanced CKD.

However, it is important to note that the term prediction or predictive performance can be misleading as it is assessed differently in literature. Some studies use the term predictive performance by correlation or survival analysis, whereas others use prediction models. The predictive performance of systolic function estimated by left ventricular EF and other conventional echocardiographic parameters like fractional shortening at the endocardial and midwall level has been investigated in asymptomatic dialysis patients. The study showed a prognostic value of EF concerning cardiovascular events [28]. In another study, left ventricular EF significantly predicted cardiovascular mortality in 1254 incident haemodialysis patients [29]. The results of these studies need to be interpreted with caution. In case of the first study the presence of concentric left ventricular hypertrophy or left ventricular remodelling, which was found in 77% of the dialysis cohort, leads to an overestimation of EF restricting its predictive power [28]. The significance of the second study results is limited by a low prevalence of left ventricular systolic dysfunction defined as an EF <50% in only 13% of the patients, as well as a low rate of cardiovascular death during follow-up (7.9% of the patients) [29].

In CKD patients, determination of the EF seems to represent a relatively insensitive method to detect preclinical stages of heart failure [26, 30, 31], which limits its power as a predictive tool. This may partly relate to the complex pathophysiology of uraemic cardiomyopathy. A common characteristic of uraemic cardiomyopathy is left ventricular hypertrophy [14, 28, 32], which is hardly recognized by EF and which leads to an overestimation of left ventricular function [10, 28, 33]. The findings mentioned above strengthen the need for sensitive clinical markers for asymptomatic individuals and raise the question whether GLS may be such a kind of marker. In 2013, Liu *et al.* assessed the prognostic performance of longitudinal strain in a cohort of 88 stable haemodialysis patients with preserved EF and found GLS to represent an independent predictor of all-cause mortality [26]. In another study, GLS was compared with EF concerning its predictive performance in advanced CKD (Stages 4, 5 and 5D). The authors found that GLS had superior predictive power compared with established cardiovascular risk factors including EF [13]. In the fraction of patients with preserved EF (≥50%), impaired GLS results heralded increased cardiovascular mortality, but there was no association with all-cause mortality. In contrast to our study design, the follow-up was much longer at 7.8 ± 4.4 years. And, while we focused on dialysis patients, Krishnasamy *et al.* [13] included less advanced CKD stages as well. Concerning the strain values they did not find a difference between dialysis and non-dialysis patients. Another study that differentiated between CKD and dialysis patients when testing different predictors of all-cause mortality found that among a wide set of echocardiographic parameters, GLS represented the only significant predictor of all-cause mortality in pre-dialysis patients and, if combined with the E/Em ratio, also in dialysis patients [20].

A recent study focusing on speckle-tracking echocardiography as a predictor of major adverse cardiac events in CKD patients included CKD patients suffering from all stages of CKD, albeit only those, who according to clinical and conventional echocardiographic parameters exhibited no evidence of pre-existing cardiovascular disease. Thus, all patients included had normal EF values. Among all parameters included in their statistical analysis, only GLS and aortic pulse wave velocity significantly predicted major adverse cardiovascular events in these asymptomatic patients [34].

When discussing the advantage of speckle-tracking echocardiography as a more sensitive predictor of cardiac events than conservative EF, technical advantages of 2D-derived speckle-tracking echocardiography should be stressed: As strain analysis is based on wall deformation, changes in the length of myocardial tissue are assessed and not wall motion as EF, such that speckle-tracking echocardiography is angle- and examiner-independent. With the help of semi-automated offline analysis, strain measurements are reproducible, much more accurate and can be obtained even by non-expert readers [35].

Our study has some limitations concerning the design as well as the technical realization. Important aspects are our relatively small cohort size and the relatively short average follow-up period of 2.1 years but this may be compensated by a relatively high rate of primary outcomes. Although the primary outcome occurred in about a quarter of our study population, this still limited the number of variables that could be included into the baseline model. Also, our strain values were based on 2D speckle-tracking echocardiography. An advanced imaging technique using 3D data sets has already been developed and this might further improve the value of speckle-tracking echocardiography. Finally, as mentioned above, the term prediction or predictive performance is used differently in many studies. The strength of our study is that we have used a range of predictive performance measures and obtained consistent results.

Future studies in larger cohorts may help to better establish GLS as a screening parameter for future cardiovascular events in patients suffering from advanced CKD.

## SUPPLEMENTARY DATA

Supplementary data are available at ckj online.

## FUNDING

This study was supported by a grant from the Dutch Kidney Foundation (16OKG12) and by SFB TRR219, project C01.

## CONFLICT OF INTEREST STATEMENT

None declared. 

## Supplementary Material

sfaa161_Supplementary_Data
