# Supplementary material for: Speckle-tracking echocardiography in comparison with ejection fraction for prediction of cardiovascular mortality in patients with end-stage renal disease
Source: Clin Kidney J. 2021 Jan 19;14(6):1579–85. doi: 10.1093/ckj/sfaa161 (PMC8280917; doi:10.1093/ckj/sfaa161)
Supplement: sfaa161_Supplementary_Data [file sfaa161_supplementary_data.docx]

Supplemental Material:

Speckle Tracking Echocardiography in comparison to Ejection Fraction for Prediction of Cardiovascular Mortality in Patients with End Stage Renal Disease

Janna Terhuerne^1^, Merel van Diepen^2^, Rafael Kramann^1^, Johanna Erpenbeck^1^, Friedo Dekker^2^, Nicolaus Marx^3^, Jürgen Floege^1^, Michael Becker^3^, Georg Schlieper^1,4^

^1^Division of Nephrology and Clinical Immunology and ^3^Department of Cardiology, Medical Faculty RWTH Aachen University, Aachen, Germany^2^; Department of Clinical Epidemiology, Leiden University Medical Center, Leiden, The Netherlands; ^4^Center for Nephrology, Hypertension, and Metabolic Diseases, Hannover, Germany

**Supplemental Figure 1**. Calibration plot representing cardiovascular death rates predicted by ejection fraction values compared to observed frequencies.

Predictor: ejection fraction

Outcome: cardiovascular mortality

**Supplemental Figure 2**. Calibration plot representing cardiovascular death rates predicted by global longitudinal strain values compared to observed frequencies

Predictor: global longitudinal strain

Outcome: cardiovascular mortality

**Supplemental Figure 3**. Calibration plot representing cardiovascular death dates predicted by variables of the baseline model compared to observed frequencies.

Predictor: baseline model (age, sex, diabetes mellitus, dialysis vintage)

Outcome: cardiovascular mortality

**Supplemental Figure 4**. Calibration plot representing cardiovascular death rates predicted by ejection fraction values added to the baseline model compared to observed frequencies.

Predictor: baseline model (age, sex, diabetes mellitus, dialysis vintage) + ejection fraction

Outcome: cardiovascular mortality

**Supplemental Figure 5**. Calibration plot representing cardiovascular death rates predicted by global longitudinal strain values added to the baseline model compared to observed frequencies.

Predictor: baseline model (age, sex, diabetes mellitus, dialysis vintage) + global longitudinal strain

Outcome: cardiovascular mortality
